# Influence of Wine on Bone Mineral Density

**DOI:** 10.3390/nu17121981

**Published:** 2025-06-11

**Authors:** Nathália Dantas Duarte, Paula Buzo Frigério, Felipe de Souza Duarte, Roberta Okamoto, Daniela Vieira Buchaim, Geraldo Marco Rosa Junior, Cleuber Rodrigo de Souza Bueno, Carlos Henrique Bertoni Reis, Rogerio Leone Buchaim, João Paulo Mardegan Issa

**Affiliations:** 1 Department of Diagnosis and Surgery, Araçatuba School of Dentistry (FOA-UNESP), São Paulo State University, Araçatuba 16015-050, Brazil; nd.duarte@unesp.br (N.D.D.); paula.frigerio@unesp.br (P.B.F.); fs.duarte@unesp.br (F.d.S.D.); 2 Department of Basic Sciences, Araçatuba School of Dentistry (FOA-UNESP), São Paulo State University, Araçatuba 16015-050, Brazil; roberta.okamoto@unesp.br; 3 Graduate Program in Anatomy of Domestic and Wild Animals, Faculty of Veterinary Medicine and Animal Science, University of São Paulo (FMVZ-USP), São Paulo 05508-270, Brazil; danibuchaim@alumni.usp.br (D.V.B.); rogerio@fob.usp.br (R.L.B.); 4 Department of Anatomy, Medical and Dentistry School, University Center of Adamantina (FAI), Adamantina 17800-000, Brazil; cleuberbueno@fai.com.br; 5 Department of Anatomy, Dentistry School, Faculty of the Midwest Paulista (FACOP), Piratininga 17499-010, Brazil; geraldo.junior@facop.com.br; 6 Beneficent Hospital (HBU), University of Marilia (UNIMAR), Marilia 17525-160, Brazil; dr.carloshenriquereis@usp.br; 7 Department of Biological Sciences, Bauru School of Dentistry (FOB-USP), University of São Paulo, Bauru 17012-901, Brazil; 8 Department of Basic and Oral Biology, Ribeirão Preto School of Dentistry (FORP-USP), University of São Paulo, Ribeirão Preto 14040-904, Brazil

**Keywords:** bone density, polyphenols, wine, antioxidants, bone repair, bone regeneration, osteoporosis, alcohol consumption, alcohol intake

## Abstract

**Background**: Considering the increasing interest in strategies to prevent osteoporosis and other bone-related conditions, it is relevant to critically assess the existing evidence on the potential benefits of phenolic compounds in wine on bone metabolism. **Objectives**: This integrative review aims to evaluate clinical and animal studies investigating the influence of wine consumption on bone mineral density (BMD). **Methods**: The search was conducted in PubMed, Scopus, and Embase databases until April 2025. The key question was: “Does wine consumption influence BMD?”. **Results**: After searching the identified databases, 108 studies were screened, and 7 were included in the final analysis. **Conclusions**: This review suggests a possible association between light to moderate wine consumption and favorable effects on BMD, particularly in the spine and femoral neck. However, these findings should be interpreted cautiously due to the predominance of observational studies. Future RCTs and systematic reviews must clarify wine’s potential role in bone health and explore non-alcoholic or low-alcohol wine alternatives with similar polyphenol content.

## 1. Introduction

Wine is an alcoholic beverage produced by the fermentation of grapes, generally composed of water (86%), alcohol (12%), glycerol and polysaccharides (1%), organic acids (0.4%), polyphenols (0.1%), minerals, and other compounds (0.5%) [1]. According to the Dietary Guidelines for Americans (2020–2025), the recommendation for wine consumption is light to moderate: a daily limit of one standard glass (150 mL; 12% ABV; ≈16.6 g/alcohol) for women and two for men, due to physiological and hormonal differences in alcohol metabolism [2]. The health benefits of wine consumption were already known to the Romans and have contributed to the widespread popularity of this beverage worldwide [3]. The benefits are associated with the presence of polyphenols [4]. These antioxidant and anti-inflammatory organic compounds are primarily found in the skins and seeds of grapes and are extracted during wine fermentation. Light to moderate wine consumption has been associated with a reduced risk of cardiovascular diseases [5], neurodegenerative protection [6], prevention of bone and metabolic disorders [7,8], as well as a lower incidence of certain types of cancer [9,10].

Red wines (e.g., Cabernet Sauvignon, Merlot, Syrah), which are fermented with grape skins, are particularly rich in flavonoids, including anthocyanins (e.g., malvidin), flavanols (e.g., catechin), flavonols (e.g., quercetin), and proanthocyanidins. These compounds contribute not only to color and taste but also to the antioxidant potential of red wine. In contrast, white wines (e.g., Sauvignon Blanc, Chardonnay), which are typically fermented without grape skins, are dominated by non-flavonoid phenolics, such as hydroxycinnamic acids (e.g., caffeic acids), benzoic acids, and stilbenes (e.g., resveratrol) [11].

The polyphenol concentration of wine is determined by several factors, including grape ripeness, terroir, vinification methods, and fermentation time [12,13]. Innovations in fermentation methods using specific yeasts and selected agricultural practices can enhance polyphenol levels [13]. The organoleptic characteristics of wine are related to the mineral composition of the vine-growing soil. For example, elevated Mn, Pb, Zn, and Cu levels have been associated with increased concentrations of resveratrol, piceid, and catechin in Cabernet Sauvignon wines. This suggests that specific soil minerals absorbed by *Vitis vinifera* grapes may affect both the quantity and type of bioactive compounds in the wine [14].

Flavonoids (90%) are the major component of the total phenolic compounds in red wine. However, it contains more concentration of resveratrol, a non-flavonoid compound, than white wine, due to extended skin and seed contact during wine fermentation [15]. A glass of red wine provides approximately 200 mg of phenolic compounds, whereas the same for white wine contains only about 40 mg [16]. For this reason, red wine is more frequently associated with health benefits, with resveratrol still being the most studied phenolic compound for bone health. This stilbene promotes bone formation and inhibits bone loss [17].

**Figure 1:**
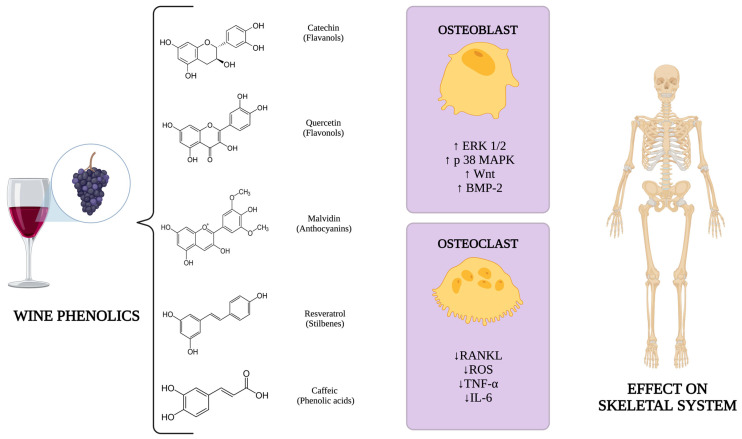
Molecular mechanisms related to wine phenolics on the skeletal system. Created with https://www.biorender.com/. Abbreviations: ERK 1/2: extracellular signal-regulated kinase 1/2; p38 MAPK: p38 mitogen-activated protein kinase; Wnt: Wnt signaling pathway; BMP-2: bone morphogenetic protein 2; RANKL: receptor activator of nuclear factor-κB ligand; ROS: reactive oxygen species; TNF–α: tumor necrosis factor alpha; IL–6: interleukin 6.

In vitro studies have demonstrated how wine phenolic compounds affect bone metabolism. These mechanisms involve the stimulation of osteoblast differentiation, maturation, and proliferation via estrogen receptors (ERs) and the activation of key signaling pathways, including ERK 1/2 [18], p38 MAPK [19], and Wnt [20]. These compounds also enhance BMP-2 synthesis [21]. In addition, polyphenols also promote osteoclast apoptosis and inhibit RANKL-induced osteoclast differentiation and the generation of reactive oxygen species [22,23]. Furthermore, they decrease the production of pro-resorptive cytokines such as TNF-α and IL-6 [24]. An overview of these molecular mechanisms is presented in Figure 1.

Considering the potential beneficial effects of phenolic compounds found in wine on bone metabolism, we hypothesized that light to moderate wine consumption may contribute to increased bone mineral density (BMD). Given the growing interest in strategies for preventing osteoporosis and other bone conditions, critically analyzing the available evidence on this topic is relevant. Therefore, this integrative review is pioneering in this field and aims to review the clinical and animal studies related to the influence of wine consumption on BMD.

## 2. Materials and Methods

This integrative review was conducted in five stages: problem identification, literature search, data evaluation, data analysis, and presentation of results, following the methodology described by Whittemore and Knafl [25].

### 2.1. Problem Identification (Key Question)

The key research question was: “Does wine consumption influence BMD?”. The inclusion criteria were clinical studies, animal studies, studies that evaluated wine isolated, and studies that performed BMD evaluation. The exclusion criteria were studies that evaluated isolated polyphenols but not wine, studies that assessed a specific diet where wine was included but without evaluating wine isolated, studies that evaluated the effect of alcohol consumption on BMD without isolating the particular impact of wine consumption, studies that did not evaluate BMD, and studies that did not address the research question. In addition, in vitro studies, reviews, theses, dissertations, and conference proceedings were excluded.

### 2.2. Literature Search

According to the eligibility criteria, searches were conducted in the PubMed, Scopus, and Embase electronic databases for studies published up to April 2025 without language, filter, or publication date restrictions. Specific search terms were used for each database. The search strategy based on combining descriptors using Boolean operators is presented in Table 1.

### 2.3. Data Evaluation and Data Analysis

Rayyan^®^ Software (https://www.rayyan.ai/) was used to manage references and remove duplicates [26]. Two independent authors (N.D.D. and P.B.F.) conducted the initial screening by reading the titles and abstracts. The full texts were reviewed to determine if the studies appeared relevant, and the inclusion and exclusion criteria were applied. A third author (F.S.D.) verified the information.

Two authors (N.D.D. and P.B.F.) collected data from the included studies for data extraction and analysis, and a third author (F.S.D.) reviewed the information. The qualitative and quantitative data collected included the author and year, study design, population size (N), sex and age of participants, as well as the type of wine and consumption details, as presented in Table 2. Additionally, the type of analysis performed, the anatomical sites evaluated, BMD values, and the reported effects on BMD are summarized in Table 3. The estimates of alcohol consumption in grams per day (g/d) are presented in Appendix A.

## 3. Results

### 3.1. Studies from Databases

**Figure 2:**
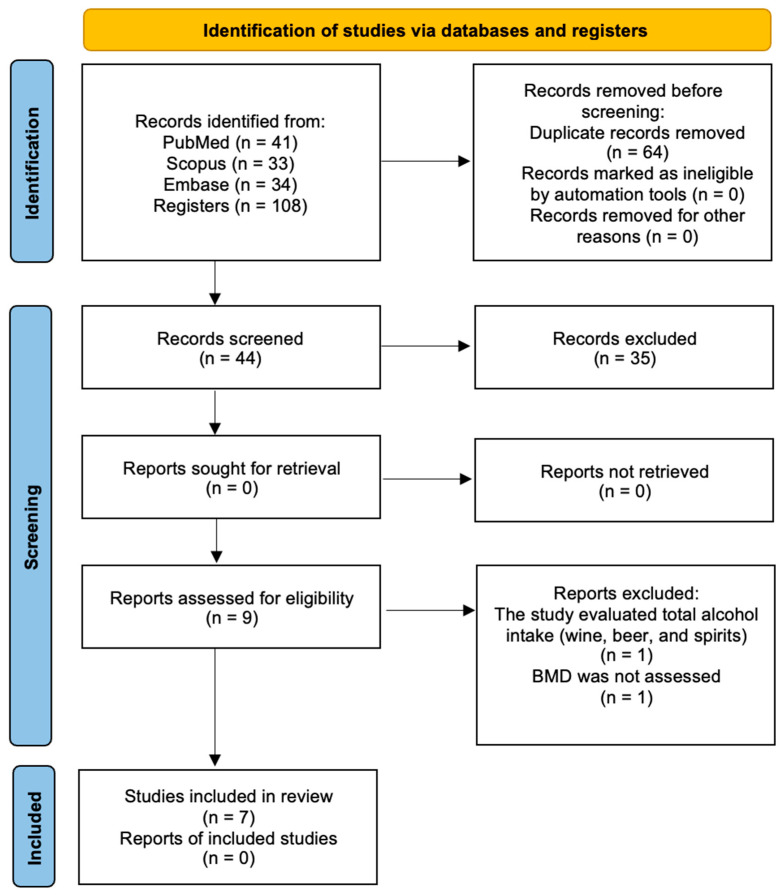
PRISMA flow diagram for the search strategy and selection process for included studies.

A total of 108 studies were identified across the previously selected electronic databases: 41 from PubMed, 33 from Scopus, and 44 from Embase. After removing 64 duplicates, 44 articles remained for title and abstract screening, and 9 were selected for a full-text assessment. Of these, 2 studies were manually excluded for not meeting the inclusion criteria: Mukamal et al. 2007 [34] evaluated total alcohol intake (including wine, beer, and spirits), and Pedrera-Zamorano et al. 2009 [35] did not assess BMD. Therefore, 7 studies were included in the final analysis. Details of the search strategy and selection process are illustrated in Figure 2.

### 3.2. Characteristics of Included Studies

The final analysis included seven studies, five clinical investigations, and two experimental animal studies. Clinical studies varied in design, such as prospective cohort studies [27,33], cross-sectional analyses [29], longitudinal follow-ups [31], and co-twin control models [30], with sample sizes ranging from 434 to 3218 participants. The populations evaluated included both men and women, predominantly middle-aged to older adults, focusing on postmenopausal women in some studies [29,33].

The consumption of wine was assessed in various formats. Some studies reported the weekly or daily frequency of consumption [27,31], while others classified intake by the number of standard drinks or glasses consumed per day [29,33]. However, the exact amount of alcohol in grams (g) or milliliters (mL) was not uniformly described across all studies. RW and WW were specifically evaluated in some investigations [27,31], while others did not specify the type of wine [29,30]. Nevertheless, it is assumed that RW was predominantly investigated, given its more frequent use in the literature.

BMD was measured primarily using dual-energy X-ray absorptiometry (DEXA), with common evaluation sites including the femoral neck (FN), hip, spine, trochanter, and total body. The findings were heterogeneous: three studies reported positive associations between moderate wine consumption and higher BMD, particularly in the spine and FN [29,30,33]; one study reported a significant association between WW intake and increased BMD, whereas RW showed no such effect [27]; and Yin et al. [31] observed that RW consumption was positively associated with spine BMD in men but not in women.

Two animal studies evaluated the effects of wine administration in rats. Cardoso et al. [28] found that low-dose daily RW intake (10 mL) resulted in improved femoral BMD in female rats, while Broulík et al. [32] demonstrated that excessive alcohol consumption with a toxic dose of 1 L of wine per day had a deleterious effect on BMD in male rats.

## 4. Discussion

In this integrative review, the available evidence supported the hypothesis that light to moderate wine consumption may contribute to increased BMD. While several studies indicated a potentially favorable effect, particularly in the spine and femoral neck, these findings were not consistent across all investigations. Moreover, excessive consumption in rats was associated with negative effects on bone density, and this toxic dose is not translatable to humans [32].

The evaluation of BMD in specific anatomical regions, such as the lumbar spine, composed of irregular bones, and the femoral neck, part of a long bone, is essential for the diagnosis and management of osteoporosis [36]. The lumbar spine is composed predominantly of trabecular bone, which is highly sensitive to hormonal and metabolic changes, making it a preferred site for the early detection of bone loss [37]. In contrast, the femoral neck consists of a combination of trabecular and cortical bone and is one of the main sites of osteoporotic fractures, particularly in postmenopausal women [38]. For the precise measurement of BMD in these sites, dual-energy X-ray absorptiometry (DXA) is the gold standard and is widely used for the diagnosis of osteoporosis and monitoring of therapeutic efficacy [39].

The Mediterranean diet (MD) is characterized by a high intake of fruits and vegetables, seeds, cereals, fish, olive oil, and moderate but regular wine intake. BMD at the lumbar spine was positively associated with the MD in a group of postmenopausal women [40]. Another dietary pattern with vegetables and wine was associated with decreased odds of having fractures in the elderly population [41]. Finally, other dietary patterns with high consumption of vegetables, seafood, seeds, and wine were directly associated with BMD at the spine and hip [42]. However, it is important to note that these findings reflect the effects of complex dietary patterns as a whole, and they cannot be associated with the observed effects of a single dietary component such as wine.

Alcohol in wine presents a paradoxical effect; while polyphenols have antioxidant, anti-inflammatory, and bone-protective properties [8], alcohol produced through the fermentation process is associated with detrimental effects on bone metabolism, especially at high doses [43]. This paradox suggests that the observed benefits may depend on a balance between the protective effects of polyphenols and the potential harms of alcohol. Therefore, chronic and excessive wine consumption may negate its positive effects, as demonstrated in the animal study included in this review, in which toxic doses of alcohol led to a reduction in BMD [32]. Moreover, it poses a risk for alcoholism, which can result in serious health consequences, mainly liver and cardiovascular diseases, and neurological disorders [44].

The effects of alcohol consumption on bone tissue are related to both the ingested dose and the duration of intake. However, the exact effects depend on factors such as age, sex, hormonal status, and the type of alcoholic beverage. When more than two glasses are consumed per day, the effects of alcohol on bone tissue become deleterious. The mechanisms responsible for these effects are that alcohol acts directly by altering the number and activity of osteoblasts and osteoclasts, as well as by increasing osteocyte apoptosis. Additionally, the observed changes may be partly modulated by the Wnt/DKK1 signaling pathway due to increased oxidative stress. Finally, alterations in cell differentiation led to low bone mass and are associated with fat accumulation in the bone marrow. In addition, the effects of alcohol on bone can also occur indirectly through reduced caloric intake and changes in body composition [43].

In light of the potential risks associated with alcohol intake, the investigation of non-alcoholic sources of polyphenols emerges as a relevant alternative. Whole grape juice, for instance, contains phenolic compounds that may confer health benefits without the deleterious effects of alcohol [43]. An animal study showed that grape juice was able to enhance bone formation through RUNX-2 upregulation and RANKL downregulation [45]. However, the study by Cardoso et al. in rats showed lower outcomes for grape juice compared to red wine and resveratrol solution, which may be explained by differences in bioavailability. Although the absorption and bioavailability of resveratrol vary among individuals, grape juice contains a lower amount of free resveratrol, suggesting reduced bioavailability when compared to the pure compound [28]. The group that consumed red wine showed higher BMD compared to the group treated with grape juice (*p* < 0.05). Therefore, red wine and the resveratrol solution demonstrated the best outcomes for BMD [28].

There is growing interest in the development of non-alcoholic or low-alcohol wine alternatives that retain a high phenolic content, allowing consumers to benefit from wine phenolics without the risks associated with alcohol [13]. Polyphenol supplementation appears to be an interesting strategy, since controlled doses of compounds such as resveratrol have shown promising results compared to wine and juice in promoting bone health in experimental and clinical models [17,28].

Although the findings of this review suggest a potential beneficial effect of light to moderate wine consumption on BMD, current evidence is not yet sufficient to support its clinical recommendation as a preventive strategy for osteoporosis. The clinical studies included in this review indicate possible sex-related differences in the effects of wine on BMD, which may be influenced by hormonal profiles. Notably, some studies demonstrated more pronounced positive effects in men [31,33], whereas in postmenopausal women, the results were more heterogeneous [27,29]. These differences could be associated with reduced estrogen levels after menopause, as many polyphenols act on estrogen receptors in osteoblasts [46], while higher testosterone levels in men are linked to enhanced bone formation [47].

In addition to the influence of wine polyphenols on bone metabolism, studies support the beneficial effects of moderate red wine consumption on cardiovascular health, mainly resveratrol and flavonoids [48,49]. The observed benefits may be attributed to improvements in lipid profiles, reduced platelet aggregation, decreased atherosclerosis, enhanced endothelial function, lower blood pressure, and increased fibrinolysis [50,51]. Moreover, polyphenols found in red wine have shown a protective effect against dementia in preclinical models of cognitive decline. However, translational clinical evidence remains inconclusive, particularly when considering the risk–benefit balance of alcohol consumption on brain health [52].

Although this is an integrative review, we briefly assessed the included studies to ensure transparency regarding their strengths and limitations, using the simplified Newcastle-Ottawa criteria. The quality of the five clinical studies was evaluated with adaptations for cross-sectional and cohort designs, considering domains such as selection, comparability, and outcome assessment. Most clinical studies demonstrated moderate quality, with potential risks of selection bias due to self-reported alcohol intake. Additionally, there was inconsistency in reporting the quantity and type of wine consumed, and none of the studies evaluated potential environmental factors. The two animal studies followed experimental protocols but lacked standardization of dosage for human equivalency. Overall, while the studies provided promising preliminary evidence, the heterogeneity in the study design and measurement methods limits the strength of the conclusions.

Furthermore, there is a scarcity of studies that specifically evaluate the isolated effect of wine on BMD, and no randomized controlled trials (RCTs) have yet assessed this approach. The lack of standardization regarding wine type, frequency, and dosage, such as a number of glasses (in milliliters), alcohol content (in grams), and the absence of declaring the alcohol content (ABV), represents a significant limitation, hindering direct comparisons between the results and their clinical application. Therefore, RCTs must be conducted to investigate the direct relation between wine consumption and BMD, which will allow for the development of systematic reviews to provide high-quality evidence regarding the potential benefits of wine on BMD. There is a need for personalized approaches and studies that consider sex and systemic conditions, such as osteoporosis, as variables in investigating the effects of wine and its bioactive compounds on bone tissue. Finally, non-alcoholic alternatives, such as whole grape juice or specific supplementation of polyphenols, should be considered, especially in populations susceptible to alcoholism or other complications related to alcoholic beverages.

## 5. Conclusions

This review suggests a possible association between light to moderate wine consumption and favorable effects on BMD, particularly in the spine and femoral neck. However, these findings should be interpreted cautiously due to the predominance of observational studies. Future RCTs and systematic reviews must clarify wine’s potential role in bone health and explore non-alcoholic or low-alcohol wine alternatives with similar polyphenol content.

## Figures and Tables

**Table 1:** The search terms used for each database.

PubMed	Scopus	Embase
(“wine”[MeSH Terms] OR “wine”[All Fields]) AND (“bone density”[MeSH Terms] OR (“bone”[All Fields] AND “density”[All Fields]) OR “bone density”[All Fields] OR (“bone”[All Fields] AND “mineral”[All Fields] AND “density”[All Fields]) OR “bone mineral density”[All Fields] OR “BMD”[All Fields])	(TITLE-ABS-KEY (wine) AND TITLE-ABS-KEY (bone AND mineral AND density OR BMD)	‘wine’ AND ‘bone mineral density’

**Table 2:** Detailed qualitative and quantitative data from all studies included, including study design, type of wine and details consumption. Abbreviations: NR: non reported, RW: red wine, WW: white wine. ^a^ Mean (quartile range).

Author and Year	Type of Study	Population (N), Sex and Age	Type of Wine	Details Consumption
Larsen et al. 2022 [27]	Clinical Prospective cohort	Men N = 1103 (59%) Age (years): 65.7 ± 7.3 Women N = 766 (41%) Age (years): 63 ± 7.5	RW and WW	Weekly 1 glass–175 mL RW–Men: 4.4 ± 5.6 RW–Women: 2.9 ± 3.5 WW–Men: 1.7 ± 3.8 WW–Women: 3 ± 4.2 Alcohol (g/w): NR
Cardoso et al. 2017 [28]	Animal Wistar rats	Female N = 50 Age (days): 90	RW	Daily 10 mL
McLernon et al. 2012 [29]	Clinical Cross-sectional	Women N = 3218 Age (years): 50–62 54.8 ± 2.2	NR	Daily >0.0–0.5 drink 787 (24.5) ^a^ >0.5–0.1 drink 580 (18.0) ^a^ >0.1 drink 425 (13.2) ^a^ Alcohol (g/d) 1.3 (0.0–5.1) ^a^
Fairweather-Tait et al. 2011 [30]	Clinical Co-twin control	Women N = 2464 Age (years): 56.3 ± 11.9	NR	Daily Alcohol (g/d) 9.2 ± 12.3
Yin et al. 2011 [31]	Clinical Longitudinal	Men N = 434 Age (years): 63.5 ± 7.6 Women N = 428 Age (years): 62.6 ± 7.2	RW and WW	Daily Men Alcohol ≤ 20 (g/d) Alcohol > 20(g/d) Women Alcohol ≤ 10 (g/d) Alcohol > 10(g/d)
Broulík et al. 2010 [32]	Animal Velaz Prague	Male N = 8 Age (months): 2	NR	Daily 7.6 g of 95% alcohol/kg = 1 L wine Alcohol mixed in water (190 mL 95% ethanol/1000 mL)
Tucker et al. 2009 [33]	Clinical Cohort	Men N = 1182 Age (years): 61.5 ± 9.3 Women Postmenopausal N = 1289 Age (years): 62.5 ± 68.1 Premenopausal N = 248 Age (years): 48.3 ± 4.7	NR	Daily 1 glass = 118 mL–13.2 g alcohol >0–0.5 glass 0.5–1 glass 1–2 glasses >2 glasses

**Table 3:** Detailed qualitative and quantitative data from all studies included, including details about BMD. Abbreviations: BMD: bone mineral density; FN: femoral neck, RW: red wine, WW: white wine. ^a^ Mean (confidence interval).

Author and Year	Analysis Performed	Evaluated Sites	BMD	Effect on BMD
Larsen et al., 2022 [27]	Dual-energy X-ray absorptiometry	Full body	Men (g/cm^2^): 1.3 ± 0.1 Women (g/cm^2^): 1.1 ± 0.1 RW (g/cm^2^): 1.21 ± 0.14 WW (g/cm^2^): 1.17 ± 0.14	RW: Negative WW: Positive
Cardoso et al., 2017 [28]	Dual-energy X-ray absorptiometry	Femur	0.175 ± 0.01 (g/cm^2^)	Positive
McLernon et al., 2012 [29]	Dual-energy X-ray absorptiometry	FN and spine	FN (g/cm^2^): Mean 0.84–0.85 Spine (g/cm^2^): Mean 1.02–1.04	Positive for FN and spine
Fairweather-Tait et al., 2011 [30]	Dual-energy X-ray absorptiometry	Hip, FN, and spine	Hip (g/cm^2^): 0.77 ± 0.12 FN (g/cm^2^): 0.87 ± 0.15 Spine (g/cm^2^): 0.95 ± 0.15	Positive for spine
Yin et al., 2011 [31]	Dual-energy X-ray absorptiometry	Hip and spine	Men Alcohol ≤ 20 g/d Spine (g/cm^2^): 1.05 ± 0.16 Hip (g/cm^2^): 1.02 ± 0.14 Alcohol > 20 g/d Spine (g/cm^2^): 1.07 ± 0.18 Hip (g/cm^2^): 1.04 ± 0.14 Women Alcohol ≤ 10 g/d Spine (g/cm^2^): 0.97 ± 0.16 Hip (g/cm^2^): 0.92 ± 0.15 Alcohol > 10 g/d Spine (g/cm^2^): 0.97 ± 0.14 Hip (g/cm^2^): 0.9 1 ± 0.11	RW: Positive for spine in men WW: Negative
Broulík et al., 2010 [32]	X-ray	Femur	1.480 ± 0.04 (g/mL)	Negative
Tucker et al., 2009 [33]	Dual-energy X-ray absorptiometry	Hip, FN, trochanter, spine	Men >0–0.5 glass Hip (g/cm^2^): 1.035 (1.022–1.049) ^a^ FN (g/cm^2^): 0.970 (0.957–0.983) ^a^ Trochanter (g/cm^2^): 0.878 (0.865–0.891) ^a^ Spine (g/cm^2^): 1.313 (1.292–1.333) ^a^ 0.5–1 glass Hip (g/cm^2^): 1.058 (1.034–1.082) ^a^ FN (g/cm^2^): 0.988 (0.965–1.011) ^a^ Trochanter (g/cm^2^): 0.902 (0.878–0.926) ^a^ Spine (g/cm^2^): 1.357 (1.320–1.394) ^a^ 1–2 glasses Hip (g/cm^2^): 1.038 (1.005–1.072) ^a^ FN (g/cm^2^): 0.961 (0.929–0.993) ^a^ Trochanter (g/cm^2^): 0.893 (0.860–0.926) ^a^ Spine (g/cm^2^): 1.314 (1.263–1.364) ^a^ >2 glasses Hip (g/cm^2^): 1.059 (1.016–1.102) ^a^ FN (g/cm^2^): 0.974 (0.933–1.015) ^a^ Trochanter (g/cm^2^): 0.910 (0.868–0.953) ^a^ Spine (g/cm^2^): 1.398 (1.333–1.464) ^a^ Postmenopausal >0–0.5 glass Hip (g/cm^2^): 0.895 (0.880–0.910) ^a^ FN (g/cm^2^): 0.853 (0.838–0.867) ^a^ Trochanter (g/cm^2^): 0.703 (0.689–0.716) ^a^ Spine (g/cm^2^): 1.132 (1.110–1.155) ^a^ 0.5–1 glass Hip (g/cm^2^): 0.904 (0.880–0.928) ^a^ FN (g/cm^2^): 0.853 (0.829–0.877) ^a^ Trochanter (g/cm^2^): 0.711 (0.689–0.733) ^a^ Spine (g/cm^2^): 1.160 (1.124–1.197) ^a^ 1–2 glasses Hip (g/cm^2^): 0.905 (0.874–0.937) ^a^ FN (g/cm^2^): 0.858 (0.826–0.889) ^a^ Trochanter (g/cm^2^): 0.704 (0.675–0.733) ^a^ Spine (g/cm^2^): 1.161 (1.113–1.209) ^a^ >2 glasses Hip (g/cm^2^): 0.938 (0.895–0.980) ^a^ FN (g/cm^2^): 0.891 (0.849–0.933) ^a^ Trochanter (g/cm^2^): 0.754 (0.715–0.793) ^a^ Spine (g/cm^2^): 1.206 (1.142–1.270) ^a^ Premenopausal >0–0.5 glass Hip (g/cm^2^): 0.988 (0.967–1.009) ^a^ FN (g/cm^2^): 0.949 (0.926–0.971) ^a^ Trochanter (g/cm^2^): 0.777 (0.757–0.798) ^a^ Spine (g/cm^2^): 1.243 (1.213–1.272) ^a^ 0.5–1 glass Hip (g/cm^2^): 1.022 (0.989–1.056) ^a^ FN (g/cm^2^): 0.981 (0.947–1.016) ^a^ Trochanter (g/cm^2^): 0.798 (0.767–0.830) ^a^ Spine (g/cm^2^): 1.273 (1.227–1.319) ^a^	Positive in postmenopausal women in all sites

## Abbreviations

The following abbreviations are used in this manuscript:

ABV	Alcohol by Volume
mL	milliliter
g	gram
g/mL	gram per milliliter
g/cm^2^	grams per square centimeter (bone mineral density)
g/d	grams per day
g/w	grams per week
L	liter
BMD	Bone Mineral Density
FN	Femoral Neck
DEXA/DXA	Dual-energy X-ray Absorptiometry
RW	Red Wine
WW	White Wine
ER	Estrogen Receptor
ERK 1/2	Extracellular Signal-Regulated Kinases 1/2
p38 MAPK	p38 Mitogen-Activated Protein Kinase
Wnt	Wingless/Integrated signaling pathway
BMP-2	Bone Morphogenetic Protein-2
RANKL	Receptor Activator of Nuclear Factor κB Ligand
ROS	Reactive Oxygen Species
TNF-α	Tumor Necrosis Factor-alpha
IL-6	Interleukin 6
RUNX-2	Runt-related Transcription Factor 2
NR	Not Reported
RCT	Randomized Clinical Trial
MD	Mean Difference
MDPI	Multidisciplinary Digital Publishing Institute
Mn	Manganese
Pb	Lead
Zn	Zinc
Cu	Copper
e.g.	for example

## Appendix A

The estimates of alcohol consumption in grams per day (g/d) are presented in Table A1, based on the wine intake data reported in the included studies. The conversion was performed using a standard glass of 175 mL with 12% ABV as a reference, which corresponds to approximately 16.6 g of alcohol per glass, and calculated according to the formula as shown in Equation (A1):

(A1) $$\mathrm{Pure}\;\mathrm{alcohol}\;(g)=\mathrm{Volume}\;(\mathrm{mL})\;\times \;(\frac{\mathrm{ABV}(\%)}{100})\times 0.789$$

Volume (mL) = the amount of wine in milliliters; ABV (%) = alcohol by volume (percentage of alcohol content); 0.789 = the density of ethanol in g/mL.

**Table A1:** Estimates of alcohol consumption in grams per day (g/d) of the included studies.

Author and Year	Estimate of Alcohol
Larsen et al., 2022 [27]	RW–Men: ≈10.4 g/d RW–Women: ≈6.9 g/d WW–Men: ≈4.0 g/d WW–Women: ≈7.1 g/d
Cardoso et al., 2017 [28]	≈0.95 g/d
McLernon et al., 2012 [29]	1.3 g/d
Fairweather-Tait et al., 2011 [30]	9.2 g/d
Yin et al., 2011 [31]	Men 20 g/d Women 10 g/d
Broulík et al., 2010 [32]	≈76 g/d
Tucker et al., 2009 [33]	13.2 g/d
